# Vertical Distribution and Composition of Plastics in Coastal Areas of the Gulf of Cádiz: Insights into Transport Dynamics

**DOI:** 10.1021/acs.est.5c03513

**Published:** 2025-08-05

**Authors:** Rocío Quintana, Sandra Manzano-Medina, Lucía Pérez-López, Amets Oyón-Sanz, Daniel González-Fernández, Juan Ignacio González-Gordillo, Elisa Marti, Fidel Echevarría, Carmen Morales-Caselles

**Affiliations:** Department of Biology, University Marine Research Institute INMAR, University of Cádiz and European University of the Seas SEA-EU, 11510 Puerto Real, Spain

**Keywords:** microplastic, plastic, vertical distribution, compartments, coastal areas

## Abstract

Plastic distribution in the surface water of the marine environment has been well-documented over the years. However, vertical distribution of plastic within the water column remains poorly understood due to a lack of in situ data. This study investigates the vertical distribution of plastic particles in coastal areas of the Gulf of Cádiz, examining the surface (0–0.2 m), subsurface (0.2–1.2 m), water column (1.2–100 m), and sediment layers. Using a high vertical resolution sampling, we analyzed plastic concentration patterns across different environmental compartments, as well as plastic particle characteristics. Our results show the highest plastic concentrations in the sediments (97.9%). Concentrations along the water column decrease exponentially with depth, retaining the particles in the mixed layer just before reaching the pycnocline. Fragment-type microplastics dominate in all compartments, while film-type and line are most prevalent below the sea surface (>0.2 m). The sediment contains the densest polymers and the smallest particle sizes, likely due to density-driven sedimentation and fragmentation processes. These findings highlight the role of vertical transport in the distribution and potential accumulation of plastics across compartments, which is crucial for understanding their fate and long-term environmental impacts.

## Graphical abstract

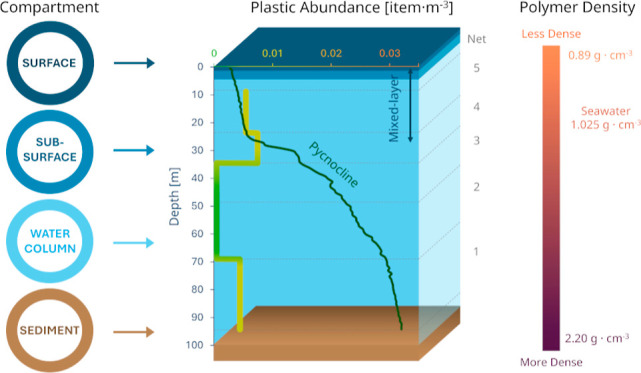

## 1 Introduction

Plastic polymers have been reported in ocean ecosystems worldwide, ranging from the deepest parts of the ocean^1^ to the highest peak on Earth.^2^ Most of the sampling effort has focused on understanding the spatial distribution of plastics on surface water.^3–5^ However, plastics do not remain on the water surface for their entire lifespan; rather, they undergo fragmentation, ingestion by fauna, wind mixing, or biofouling^6–10^ often resulting in their removal from the surface.

The distribution of plastic particles along the water column is poorly understood due to the complexity of controlling processes and limited availability of comprehensive in situ data.^11^ Most existing studies rely on numerical models to simulate vertical transport,^12–14^ while sinking rates of plastic particles are usually estimated from laboratory experiments.^15,16^ Plastic concentrations in the water column are often extrapolated from the surface measurements,^6,17^ which may potentially lead to overestimations.^18^ Although some studies have provided valuable in situ data that have significantly contributed to our understanding of vertical plastic transport,^19–28^ these efforts are predominantly focused on the open ocean and differ widely in sampling designs, depth resolution, sampled volumes, and instrumentation used (e.g., nets, pumps, in situ filtration).

Many of the plastic particles sink vertically through the water column to the bottom, where they can be retained in the sediment for long periods.^29^ Hence, the importance of understanding the concentrations of plastics in sediment is that it can provide insights into the fate and long-term patterns of deposition and accumulation that are not evident from measurements in the water column.

It is known that nearshore areas concentrate large amounts of marine plastic pollution due to their proximity to land-based waste inputs;^30,31^ however, surveys to simultaneously measure plastic concentrations in different nearshore environments (i.e., surface water, water column, and sediment) are rare. The concentrations of plastic widely vary along the nearshore areas according to location near an urban center or other hotspots,^31,32^ the presence of rivers or estuaries,^33–35^ and the dynamics of current circulation,^36,37^ among others.

In this context, understanding the multienvironment distribution of plastic in nearshore areas is essential to comprehend the connection between pollution sources and potential risks. The present study addresses these critical gaps by presenting a high-resolution vertical profiling of plastic from surface waters to sediments in coastal areas of the Gulf of Cádiz (GoC), covering one area near an urban center (Bay of Cádiz) and another one close to a large river mouth (Estuary of the Guadalquivir River, full river basin size approximately 57,000 km^2^). The purposes of the work are (1) to analyze the distribution of plastics across different nearshore compartments; (2) to investigate the physical drivers controlling the vertical distribution of plastic particles through the water column; and (3) to examine how plastic composition and characteristics, such as the shape, size, and density, might influence its dispersal in the ocean.

## 2 Material and Methods

### 2.1 Study Area and Sampling

The Gulf of Cádiz (GoC), located in the southwest of the Iberian Peninsula, was the focus of the PLAn (*Plastics in the Andalusian Coast*) research cruise. Conducted aboard the Spanish R/V UCADIZ, the campaign took place in October 2021, sampling locations adjacent to the mouth of the Guadalquivir estuary (G) and the Bay of Cádiz (C) (Figure 1). A total of 20 locations were sampled during this oceanographic campaign. The survey included six transects, each of them including three sampling locations at an increasing distance from shore: “shallow”, “intermediate”, and “deep” waters, apart from one transect where only one site (C7) was possible to sample due to weather conditions. Additionally, five deeper water samplings were also performed (MC1, MC2, MC3, MG4, and MG5).

**Figure 1:**
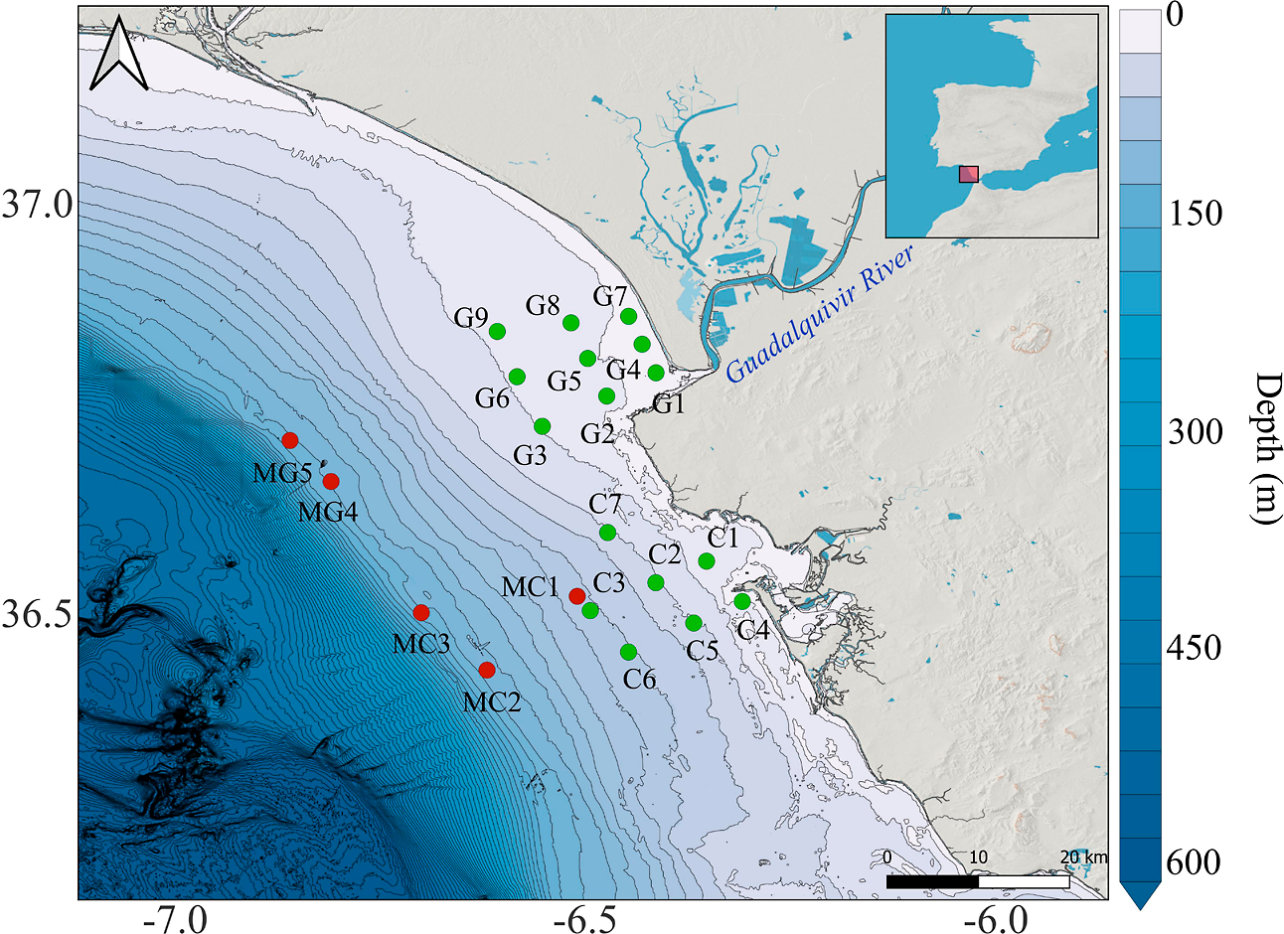
Map of the study area and the main sampling points of the PLAn campaign near the Guadalquivir estuary and in front of the Bay of Cádiz. The green dots represent the sampling using neuston net, bongo net, and box corer, and the red dots represent the sampling using neuston net and Multinet.

For the sampling points C1–C7 and G1–G9, surface, subsurface, water column, and sediment samples were obtained using a neuston net (comprising three nets: the first for surface sampling, the second and third for subsurface sampling), a bongo net (consisting of two nets), and a box corer (Table 1). Meanwhile, for the sampling points in deeper waters (MC1-MG5), the neuston net and a multiple plankton sampler (Multinet) were used to achieve a complete vertical profile (Figure S1 and Paragraph S1, Supporting Information). The first net of the Multinet (Net 1) represents an integration across the entire water column, while the remaining five nets correspond to specific depth ranges (Net 2–6). All nets used in this campaign had a mesh size of 200 μm. A total of 24 samples from the Multinet, 55 samples from the neuston net, 30 samples from the bongo net, and 48 samples from the box corer were taken during the campaign. Water samples were preserved in 4% formalin, while sediment samples were stored at 4 °C until further analysis.

**Table 1:** Sampling Methods of Each Environmental Compartment

Sampling Points	Instrument	Nets	Compartment
G1–G9; C1–C7	Neuston Net	1	Surface
		2–3	Subsurface
	Bongo Net	1–2	Water Column
	Box Corer		Sediment
MC1–MG5	Neuston Net	1	Surface
		2–3	Subsurface
	Multinet	1–6	Water Column

### 2.2 Plastic Extraction and Analysis

Once in the laboratory, samples were extracted under a fume hood by using a 200 μm mesh size sieve to separate the solid content from the 4% formalin. The sample contents were rinsed several times with filtered seawater and deposited in a crystallizer dish for further observation under the binocular lens. The density difference between the seawater and the suspected plastic particles facilitated the individual extraction by hand using tweezers. The identification of potential plastic particles was based on the methodology described by Lusher et al.^38^

The extraction of plastic from sediment samples was conducted following the methodology described by Coppock et al.^39^ A sediment-microplastic isolation (SMI) unit was used with a saturated solution of sodium chloride (NaCl), which allowed the separation of plastic by the density difference. While the NaCl solution (density ∼1.2 g/cm^3^) primarily recovers low-density polymers, recovery efficiency can also depend on particle shape, size, surface properties, and biofouling state. Thus, although some high-density or biofouled microplastics may sink and be under-represented, others may remain suspended and be captured during the separation.

For each SMI, 50 g of dry sediment and 800 mL of saturated solution were stirred for 5 min, followed by a 1 h sedimentation period. This process was repeated two more times in order to have a total of 150 g of dry sediment analyzed per replicate. After sedimentation, the upper part of the SMI was transferred into a crystallizer dish, and suspected plastic particles were manually extracted, following the same procedure used for water samples.

After all of the suspected plastic particles were removed from the samples, they were placed into a Petri dish for subsequent image analysis using the *ImageJ Fiji* software. Photographs were taken using a Nikon D810 high-resolution camera (42 Mpx) with a high contrast for the characterization of the particle (count, area, and feret). The particles were classified into four categories according to their shape: fragment, film, lines, and foam (Figure S2). Likewise, with the feret diameter obtained from ImageJ Fiji, plastic particles were classified into three size categories: microplastics (200 μm to 5 mm), mesoplastics (5–25 mm), and macroplastics (>25 mm). All microplastics smaller than 200 μm were excluded from this study to ensure a size range comparison between different compartments.

### 2.3 Quality Control

Cotton laboratory coats and nitrile gloves were used throughout the extraction process and particle identification. Prior to opening the samples, all working surfaces (including the fume hood) were cleaned with 96% ethanol. All instruments were rinsed with filtered distilled water before each use to minimize contamination between the samples. Since fibers were not included in this study, airborne contamination controls were not required.

Procedural blank controls were implemented for only sediment samples to assess complete sediment removal from the SMI unit and to identify potential contamination sources from the same unit. A total of 49 procedural blanks were conducted—one after each replicate (Figure S3)—by conducting the entire extraction process without a sample, using only sodium chloride (NaCl). The procedural blanks showed an average contamination of 0.041 ± 0.198 item·blank^–1^ (mean ± SD). This value was not corrected in the final data set, as it was considered to have a negligible influence on the overall results.

Additionally, contamination originating directly from the SMI unit was evaluated. During agitation of sediment samples within the unit, contact between the sediment and the internal surface may cause the release of PVC fragments from the unit itself. To assess this source of contamination—which was not observed in the procedural blanks—fragments were collected from various components of the SMI unit and analyzed using FTIR spectroscopy. A strong spectral match confirmed that 4.2% of the particles detected in the sediment samples originated from the SMI unit. These particles were therefore classified as procedural contamination and excluded from the final data set.

### 2.4 FTIR Analysis

Plastic polymers were identified with a PerkinElmer Spectrum3 FT-IR Spectrometer with a GladiATR Vision accessory. The scanners range from 4000 to 650 cm^–1^, with a nominal resolution of 4 cm^–1^. For each analysis, 32 scans were averaged for the background, and 4 scans were averaged for each sample. Infrared spectra of the sampled particles were compared with reference spectra from the supplier’s libraries (PerkinElmer) using SpectrumIR software. The match between spectra was accepted when the HQI (Hit Quality Index) was greater than or equal to 0.7 (94.5%), was reviewed when the score was between 0.6 and 0.7 (4.7%), and was rejected when it was less than 0.6 (0.8%).^40^ All items were inspected prior to rejection; only those with an HQI value below 0.6 were excluded from further classification (less than 1%). In total, 293 particles were analyzed, representing 100% of the particles extracted and counted from all sampled compartments. Of the analyzed particles, 87.1% were classified as plastic, 10.2% as nonplastic, and 2.7% remained unidentified. Only particles confirmed as plastics were included in the calculation of plastic concentrations for all compartments. The analysis revealed a total of 81 plastic particles from the surface, 67 from the subsurface, 40 from the water column, and 65 from the sediment.

### 2.5 Data Analysis and Statistics

Plastic concentrations in water samples were calculated using the following equation

$$C_{p}=\frac{n}{V}$$

where *C*_p_ is the plastic concentration (items·m^–3^), *n* is the number of plastic items found in each sample, and *V* is the volume filtered by each net (m^3^). For the neuston net (surface and subsurface) and bongo net (water column), the filtered volume was estimated using a mechanical flowmeter (General Oceanics) and the net opening area. For the multinet (water column), the volume was determined using two electronic flowmeters integrated in the unit: one inside the frame to determine the volume filtered and another one outside the opening (differences in flow measurement would indicate non-isokinetic sampling, e.g., by clogging).

Plastic concentration in sediment samples was calculated by weight (items·kg^–1^), area (items·m^–2^), and volume (items·m^–3^), using the equations provided in Paragraph S2. All sampled volumes and weights used for the calculations across compartments are detailed in Tables S1–S4. It is important to note that when *n* = 0, this may reflect extremely low plastic concentrations (i.e., fewer than one particle in the volume or weight sampled) rather than a true absence.

The relationship between plastic concentrations and environmental variables was assessed using Spearman’s rank correlation, as the data did not meet the assumption of normality (Shapiro–Wilk test, *p*-value < 0.05). Additionally, differences in plastic concentrations between depth layers (within and below the mixed layer) were evaluated using the Mann–Whitney–Wilcoxon test, both for the entire data set and within each study area (Cádiz and Guadalquivir). This analysis was conducted exclusively with multinet samples to avoid bias from floating plastics collected with the neuston net.

Furthermore, plastic composition (shape, size, and polymer) in relation to distance was assessed using the Kruskal–Wallis test. Given the presence of more than two groups, pairwise comparisons were performed using the Mann–Whitney–Wilcoxon test with Bonferroni correction. All statistical analyses were carried out in R (v. 4.5.0), and significance was set at *p*-value < 0.05.

For the quantification of the water column stratification, the Brunt–Väisälä frequency was implemented (*N* ^2^;^41^)­

$$N^{2}=-\frac{g}{\rho }\times \frac{d\rho }{dz}$$

where *g* is the acceleration due to gravity, ρ is the density of the seawater, and *z* is the corresponding depth in meters.

To estimate the accumulation of plastic particles in different compartments, the concentrations were normalized by the corresponding depth range of each compartment (Paragraph S3). Finally, to better understand the vertical distribution of plastics between compartments, the settling velocity of plastic particles was calculated using the model of Yu et al.;^42^ the corresponding equations are detailed in Paragraph S4.

## 3 Results and Discussion

### 3.1 Spatial Distribution: Surface and Subsurface Water

Plastic was found in all of the sampling sites in the Gulf of Cádiz (GoC). Mean concentrations were higher in the surface (0.055 ± 0.079 item·m^–3^) than in the subsurface (0.007 ± 0.009 item·m^–3^) compartments (Figure 2A,B), with a difference of 1 order of magnitude. Differences were also observed between the Cádiz and Guadalquivir areas, with an average concentration of 0.083 ± 0.109 item·m^–3^ and 0.033 ± 0.039 item·m^–3^ in the surface and 0.012 ± 0.012 item·m^–3^ and 0.004 ± 0.004 item·m^–3^ in the subsurface, respectively. Maximum concentrations were measured in surface water at sampling points C3 (0.318 item·m^–3^), G8 (0.109 item·m^–3^), and C1 (0.091 item·m^–3^).

**Figure 2:**
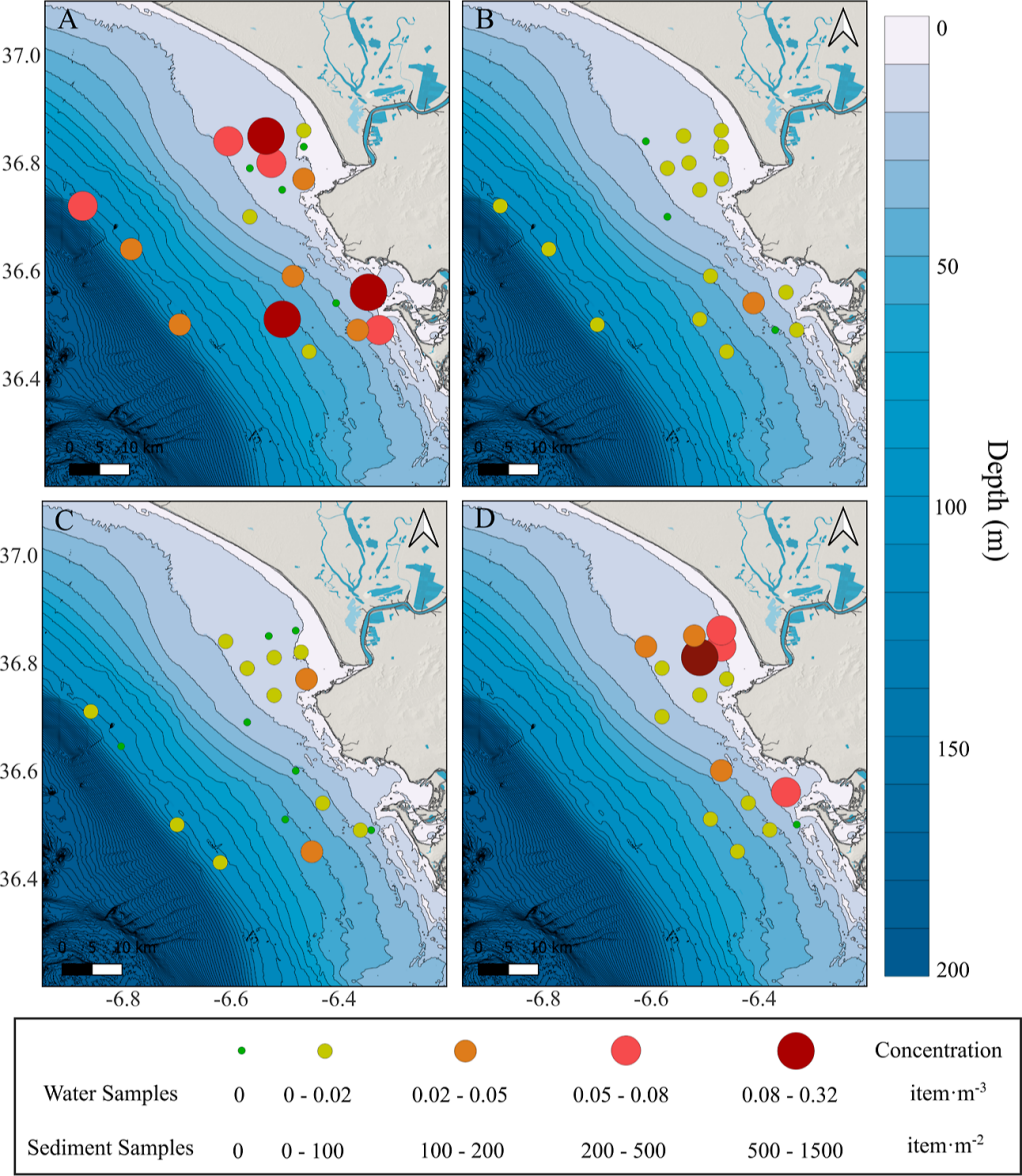
Spatial distribution of plastic concentration in (A) surface water (0–0.17 m); (B) subsurface water (0.17–1.17 m); (C) water column in shallow waters (minimum depth: 4 m; maximum depth: 45 m) and deeper waters (Net 1: MC2: 0.1–85.3 m; MC3: 1.5–94 m; MG4: 3.2–97.1 m; MG5: 35.9–98 m); and (D) sediment (minimum depth: 9 m; maximum depth: 55 m). Water sample concentration (A–C) is in item·m^–3^ and the sediment samples concentration is in item·m^–2^.

Previous studies have generally reported a decline in the microplastic concentration with increasing distance from shore as they moved further offshore^43,44^ due to the common presence of large pollution sources on land. But also, plastic inputs from rivers and estuaries are highly variable since they are affected by multiple factors such as tides, river flow, dams, rain, or storms.^31,45^ For instance, a study by González-Ortegón et al.^34^ in the GoC found that microplastic concentration in subsurface water (5 m depth) was highest near the Guadalquivir (130.5 mg·m^–3^) and Guadiana (64.6 mg·m^–3^) rivers, compared to the continental shelf (30.5 mg·m^–3^). However, in this study, no clear spatial trend was observed, either in the surface (ρ = 0.095; *p*-value = 0.727) or subsurface (ρ = −0.400; *p*-value = 0.124).

Plastic concentration in estuaries can change orders of magnitude in a very limited time range due to these factors.^46,47^ In the present study, plastic concentrations were relatively low near the river mouth, which could be related to the very low levels of river freshwater flow regulated by a dam at the head of the estuary (mean flow of 6.2 m^3^·s^–1^ during the month of October, with minimum values of 2.0 m^3^·s^–1^ and maximum values of 50.9 m^3^·s^–1^; https://www.chguadalquivir.es/saih/).

### 3.2 Vertical Distribution: Water Column

#### 3.2.1 Vertical Profiles in Shallow Waters

Plastic concentrations obtained with the bongo net correspond to a specified depth of the water column (4–45 m) (Table S2). Average concentrations in the water column were similar in both areas, obtaining 0.007 ± 0.010 item·m^–3^ in Cádiz and 0.006 ± 0.007 item·m^–3^ in the Guadalquivir area. Comparing the concentration data of surface, subsurface, and water column (Figure 2A–C), a higher concentration is observed in surface waters, with a difference of 1 order of magnitude between compartments.

In general, the plastic concentrations across the three vertical compartments were not correlated. The absence of correlation (Table S5) likely reflects the influence of hydrodynamic processes within the water column. This is particularly evident in the Guadalquivir Estuary, a tidally dominated system where tidal mixing and reduced freshwater input during periods of low river flow led to a well-mixed water column.^48^

Di Mauro et al.^49^ also reported higher surface microplastic concentrations compared to the water column of the Gulf of Mexico, using the same sampling method. The bongo net remains the most commonly used instrument for analyzing microplastics in the water column, providing integrated data across specific depth layers.^50^ Indeed, a layer-by-layer analysis of the water column can provide a more comprehensive understanding of the distribution of the plastic particles.

#### 3.2.2 Vertical Profiles in Deeper Waters

Vertical profiles showed that plastic concentrations were highest in the surface layer (0–0.17 m), with values ranging from 0.3 to 0.03 item·m^–3^ (Figure 3). As depth increased, plastic concentration decreased up to 1 order of magnitude, with some samples where no anthropogenic particles were identified (Table S3). In the Cádiz area (MC1, MC2, and MC3), the profiles had similar patterns: maximum concentration in the surface layer and a decrease in concentration between 1.17 and approximately 40 m with a mean of 0.007 item·m^–3^. The MC1 profile, being the shallowest (with the first net opening at 40.8 m), was the only profile that showed concentrations in all depth ranges sampled by the Multinet. In the MC2 profile, as depth increased (40 m onward), the concentration reached zero. Lastly, in the MC3 profile, the concentration also decreased to zero between 34.1 and 68.9 m, but an increase in plastic concentration was shown between 69 and 94 m (0.003 item·m^–3^). Regarding the profiles of the Guadalquivir, MG4, and MG5 showed clear differences. The maximum concentration was measured in the surface layer, but after the first meter of depth, the plastic concentration decreased sharply until it reached zero. This concentration increased again at certain specific depth ranges for the MG4 profile, where an increase of up to 0.008 and 0.007 item·m^–3^ was observed in the ranges from 9 to 24.6 m and from 35.4 to 51.7 m, respectively.

**Figure 3:**
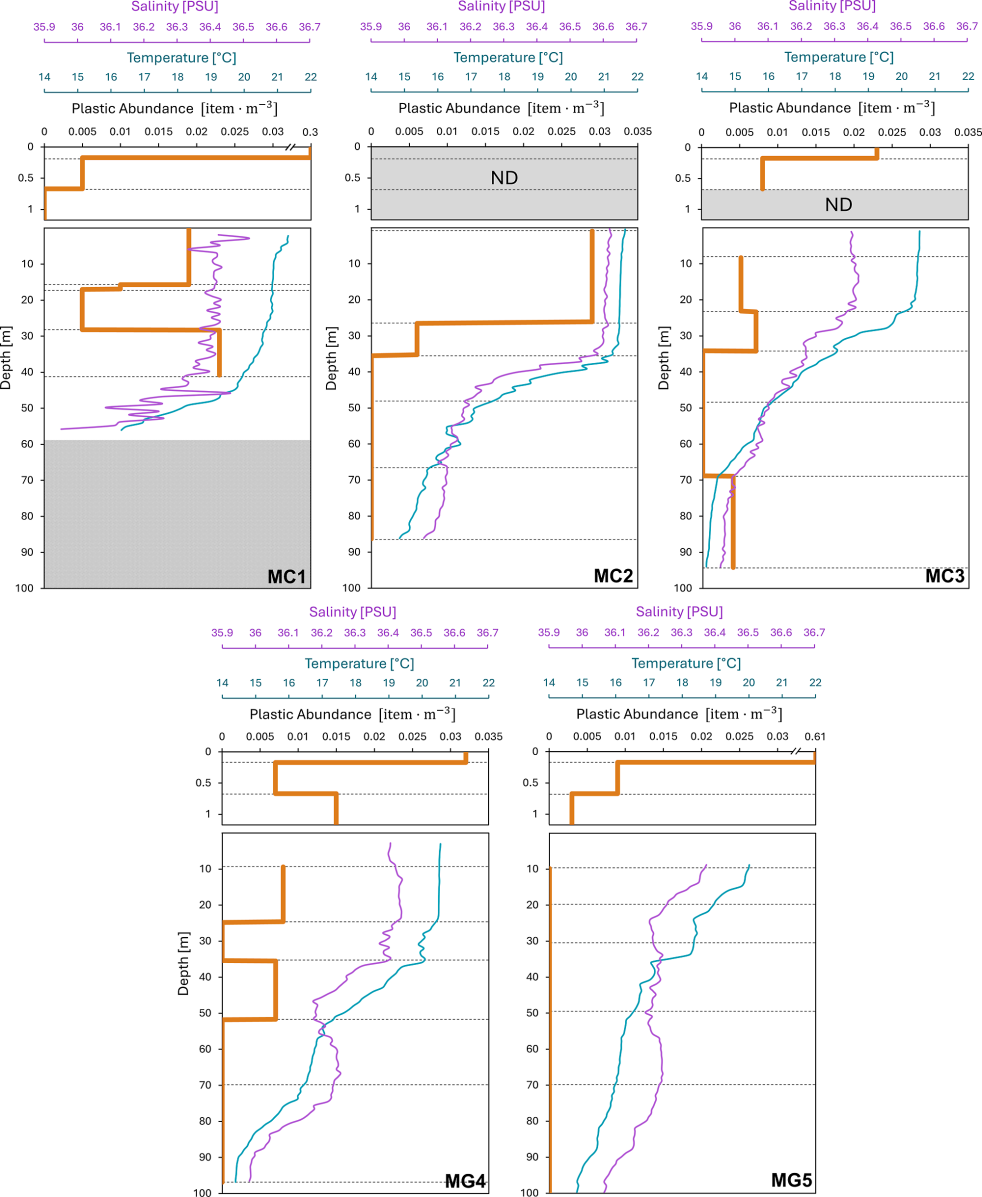
Vertical distribution of microplastic (orange lines), temperature (blue lines), and salinity (purple lines) in the water column and surface of the Cádiz (MC1, MC2, and MC3) and Guadalquivir area (MG4 and MG5). Gray dotted lines represent the different net openings of the neuston net and Multinet. For the MC1 profile, the gray box represents the bottom.

Plastic concentrations in the water column were comparable for both Multinet (Figure 3) and bongo net (Figure 2 C) samples, ranging in the same order of magnitude (Multinet mean value: 0.005 ± 0.007 item·m^–3^; bongo net mean value: 0.007 ± 0.008 item·m^–3^). A limited number of studies have documented the vertical distribution of plastic in the water column using a Multinet and have mainly focused on the open ocean. Egger et al.^24^ investigated the distribution of microplastics (0.5–5 mm) in the North Pacific Ocean, reporting concentrations lower than 0.001 item·m^–3^. A similar study conducted by Egger et al.^22^ in the North Atlantic Ocean found concentrations ranging from 0.01 to 0.001 item·m^–3^ at depths greater than 5 m. In the South Atlantic Ocean, Zhao et al.^25^ found concentrations ranging from 0 to 0.011 item·m^–3^ for microplastics between 0.3 and 5 mm. All of these studies reported comparable concentrations in the water column, given the similar size range of sampled particles.

However, upon comparison of our results with other studies that employed different sampling methodologies (sampling volumes) and size ranges, a wide variation in concentrations can be observed. Li et al.^26^ used an in situ filtration device and reported concentrations ranging from 0.2 to 2.0 item·m^–3^ in the West Pacific Ocean and 0.2–3.5 item·m^–3^ in the East Indian Ocean (size range: 0.03–6.33 mm). Similarly, Choy et al.^51^ reported higher concentrations in the Pacific Ocean, ranging from 2.9 to 15 items·L^–1^ for particles between 0.1 and 5 mm. These concentrations are several orders of magnitude higher than those reported in net-based studies, underscoring the critical influence of methodological factors, particularly the sampling depth, instrument type, sampled volume, and minimum plastic size considered.

### 3.3 Physical Properties and Vertical Plastic Distribution Analysis

The vertical distribution of plastic in the water column depends on external forces (e.g., wind, density profile, water flow, and turbulence) and plastic characteristics (e.g., density, size, and shape). Figure 3 shows the plastic concentration but also salinity and temperature profiles as a function of depth. In the Cádiz area, temperature (21–22 °C) and salinity (36.4–36.5 psu) remained stable from the surface to 30–40 m. Beyond this depth, temperature dropped by 6 °C and salinity by 0.5 psu down to 60–70 m, reaching the thermocline and halocline. Beyond this transition zone, the profiles exhibited a slight decrease with depth down to approximately 100 m. The distribution of plastic seemed to be affected by the variation of salinity and temperature,^52^ which are directly related to seawater density (Figure 4), with higher concentrations observed in the mixed layer.

**Figure 4:**
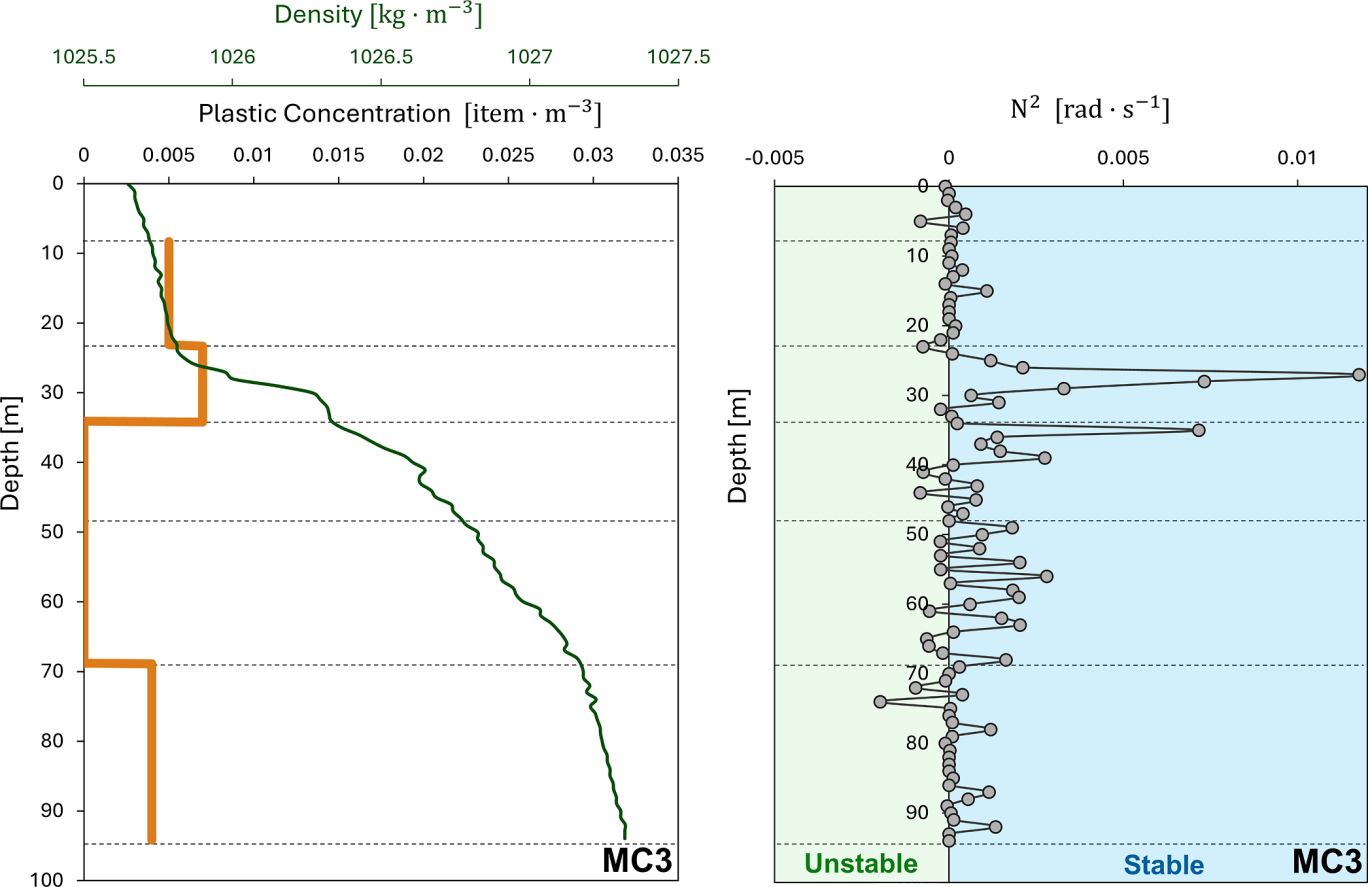
Vertical distribution of microplastic and density in the water column of the MC3. Brunt–Väisälä frequency (*N* ^2^) from the Multinet CTD. Black lines represent the different net openings of the MC3.

The Brunt–Väisälä frequency (*N* ^2^) was used to test our previous hypothesis. This variable is commonly used for describing the stratification stability of a fluid.^53^ *N* ^2^ was calculated from the density data of Multinet CTD (MC3). Throughout the first 25 m of depth, the values were close to 0 rad·s^–1^, indicating unstable stratification and, consequently, a zone of vertical mixing where the water column was homogenized (Figure 4). From 25 to 40 m depth, there was an increase in *N* ^2^ reaching a maximum of 0.012 rad·s^–1^, showing a stable stratification with a strong density gradient as depth increases. Subsequently, from 40 to 70 m depth, *N* ^2^ values decreased, approaching values near 10^–4^ rad·s^–1^, suggesting a weak stratification. From 70 to 95 m depth, the values were 0 rad·s^–1^, indicating again a vertical mixing. These density gradients could explain the presence and absence of plastics in the mixed layer and the subsequent pycnocline. Where the pycnocline could act as a density barrier due to the constant density changes within a limited depth range and, therefore, could impede the sinking of plastics further in depth.^19^ In the MC3 profile, a slight increase in plastic concentration was observed in net 5 (depth range: 23.1–34.2 m), where the stratification started at 25 m depth. This depth would act as a density barrier where plastics would tend to considerably slow down their sinking velocity, leading to their subsequent accumulation due to the constant density changes as we go deeper in the water column. Uurasjärvi et al.^54^ found similar patterns in the stratified waters of the Baltic Sea, where sinking rates and the accumulation of microplastics are affected by stratification. Similarly, Bagaev et al.,^55^ reported higher concentrations just above the pycnocline. Meanwhile, Fischer et al.^17^ reported that seasonal effects could also impact the vertical distribution of plastics, particularly during spring blooms, when particles would sink below the depth of the mixed layer due to biofouling; the rest of the year, plastic would remain within the mixed layer primarily affected by physical processes such as wind-induced mixing. This study matched our observations, as the samples were obtained in early fall, indicating that the water column would be predominantly influenced by physical processes, deepening the thermocline while the stratification is still present to act as a barrier, leading to higher concentrations of microplastics within the mixed layer rather than below it. Supporting this, the Mann–Whitney–Wilcoxon test comparing plastic concentrations inside and below the mixed layer in the Cádiz area showed significantly higher concentrations in the upper layer (*p*-value = 0.0089).

The vertical profiles of the Guadalquivir area (MG4 and MG5) showed patterns different from those of Cádiz. In the MG4 profile, constant salinity (36.4 psu) and temperature (20.5 °C) were observed from the surface to 30 m. The thermocline and halocline were placed below this depth. The thermocline, exhibiting a temperature gradient of 6°, extended down to 90 m. Meanwhile, the halocline ended at 50 m but underwent a slight increase up to 70 m before decreasing again. For the MG5 profile, salinity decreased from 10 to 25 m and then remained constant (36.2 psu) throughout the entire water column. Meanwhile, the temperature consistently decreased from 10 to 100 m depth, with a difference of 5°. For both profiles, it was challenging to establish a relationship between the physical variables and the concentration, likely due to the influence of river inputs (estuary under the tidal regime). No significant differences were observed between the concentrations inside and below the mixed layer (Mann–Whitney–Wilcoxon test, *p*-value = 0.761). Moreover, the plastic distribution in the water column may not be solely explained by vertical processes, as horizontal advection could play a major role. This is particularly relevant in coastal areas affected by river inputs, such as our study site, where such advection processes may contribute to the redistribution of plastics. The clearest conclusion was that the surface layer showed higher concentrations in all profiles.

### 3.4 Sediment Plastic Distribution

The average concentration of plastic in sediment was 196.8 ± 355.0 items·m^–2^ or 9.0 ± 10.6 items·kg^–1^ (Figure 2D). No correlation was found between the sediment and the other compartments nor with the distance to the coast. However, significant differences in plastic concentrations were observed in different zones. The Guadalquivir area showed concentrations almost three times higher (275.9 ± 458.3 items·m^–2^ or 11.4 ± 13.4 items·kg^–1^) than in the Cádiz area (95.0 ± 116.1 items·m^–2^ or 6.0 ± 4.6 items·kg^–1^). This finding could be related to the influence of the long-term input of plastic particles from the Guadalquivir River. Estuaries, particularly intertidal and bottom pools, act as an important sink for microplastics.^56^ It remains less clear whether these areas act as permanent or temporary sinks, due to the resuspension and remobilization by turbulence or bioturbation.^57^

Relevant high concentrations were measured at G5 (1484.1 ± 1338.5 items·m^–2^ or 46.7 ± 37.1 items·kg^–1^), G7 (256.6 ± 314.5 items·m^–2^ or 11.1 ± 13.9 items·kg^–1^), and G4 (208.2 ± 360.6 items·m^–2^ or 6.7 ± 11.5 items·kg^–1^), which may be attributed to a localized sediment deposition. This result agrees with the study by Rodríguez-Ramírez et al.,^58^ which reported increasing sedimentation rates in this particular area. Several authors have reported higher concentrations of microplastics in the bottom sediment of estuaries compared with the neighboring environment. In the Río de la Plata Estuary (Argentina), the average concentrations of microplastics in sediment (613 items·m^–2^) were six times higher than in the water column.^59^ Willis et al.^60^ also found higher concentrations of microplastics in sediments in the Derwent Estuary, Tasmania (Australia). This confirms the suitability of sediments for plastic monitoring purposes.

### 3.5 Plastic Composition in Different Compartments (Polymer, Shape, and Size)

Plastic composition plays an important role in their distribution, as the material density, size, and shape relate to changes in the advective velocity of the plastic particle.^36^ In our study, the most abundant polymers were polyethylene (PE) with 62.2% and 54.4% and polypropylene (PP) with 23.7% and 30.4% for surface and subsurface waters in the Cádiz and Guadalquivir areas, respectively (Figure 5). These low-density polymers tend to float at the surface and are the most common polymers found in the marine environment.^8,34^ Accordingly, most plastic waste is made of PE and PP. High-density polymers (e.g., polyvinyl acetate (PVA), polyethylene terephthalate (PET), and polyvinyl chloride (PVC)) were also found in surface and subsurface waters, but in relatively smaller proportions. Suaria et al.^4^ identified similar plastic composition in the surface waters of the Mediterranean Sea, with a higher proportion of PE and PP and a lower proportion of high-density polymers (PVC, PVA, and PET).

**Figure 5:**
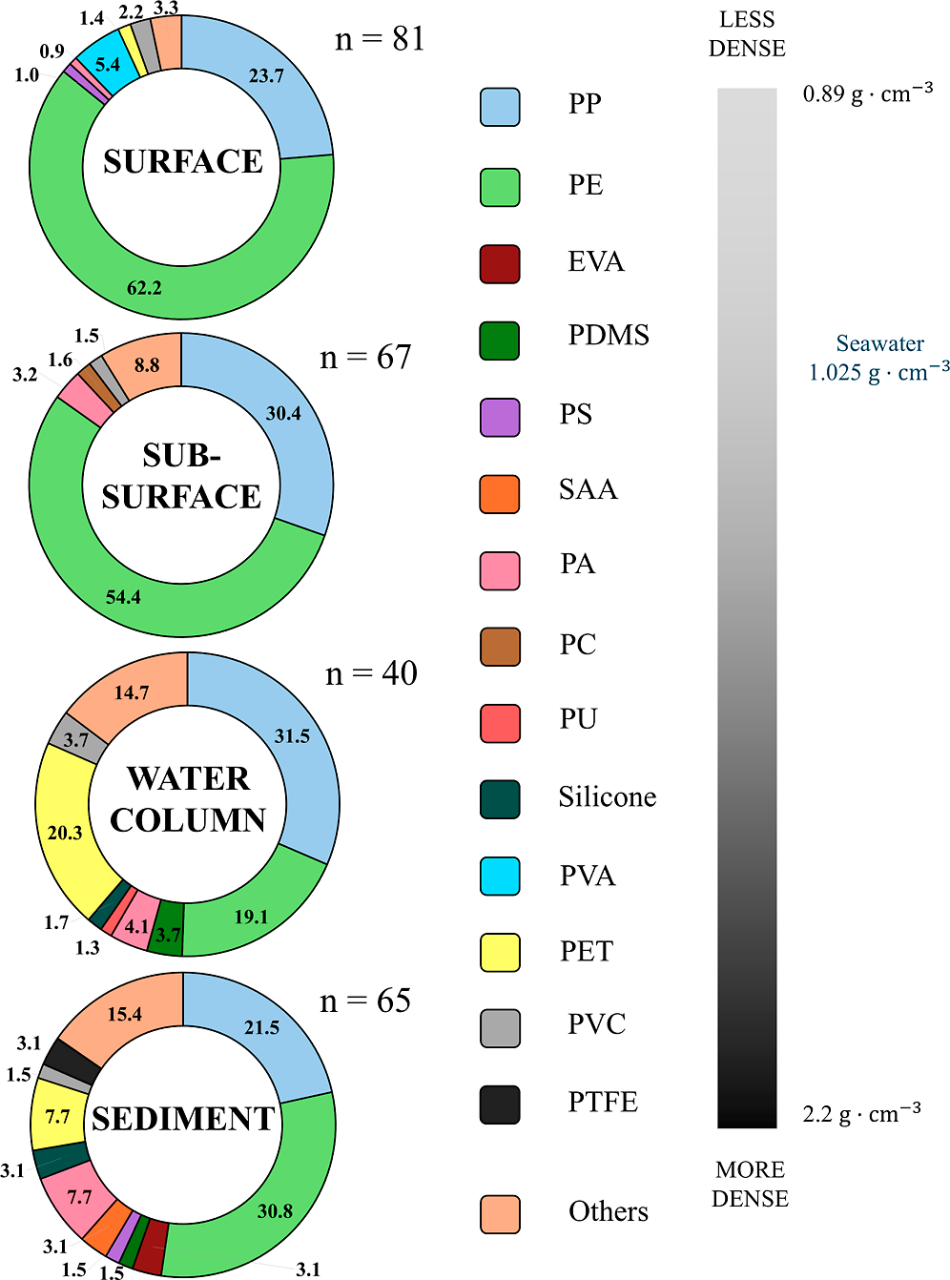
Plastic composition in surface, subsurface, water column, and sediments. Polymers are ordered from lowest to highest density: polyethylene (PE), polypropylene (PP), ethyl vinyl acetate (EVA), polydimethylsiloxane (PDMS), polystyrene (PS), styrene allyl alcohol (SAA), polyamide (PA), polycarbonate (PC), polyurethane (PU), silicone, polyvinyl acetate (PVA), polyethylene terephthalate (PET), polyvinyl chloride (PVC), and polytetrafluoroethylene (PTFE). Others include poly­(ethyl acrylate) (PEA), copolymer, poly­(vinyl stearate) (PVS), melamine urea formaldehyde (MUF), rubber, styrene butadiene copolymer (SBC), and acrylonitrile butadiene styrene-polyvinyl chloride (ABS-PVC). Numbers show percentage.

In the water column, PE decreased its proportion with depth, from 62.2% at the surface to 19.1% in the water column, whereas PP remained approximately the same percentage (31.5%) in comparison with the surface and subsurface (Figure 5). The water column showed a higher diversity of plastic polymers, including polydimethylsiloxane (PDMS), polyamide (PA), and polyurethane (PU), but there was also an increase in the high-density polymers such as PET (20.3%) and PVC (3.7%). The reduction in the proportions of PE and PP is also reported in other studies. Erni-Cassola et al.^9^ found low-density polymers (such as PP and PE) decreasing in abundance through the water column, whereas denser polymers increased with depth.

Plastic compositions in the sediment showed the lowest abundance of PP (21.5%) but a slight increase in PE (30.8%). This finding has been reported in other studies that have identified low-density microplastics in sediments.^61^ Meanwhile, denser polymers such as PVC (1.5%) and PET (7.7%) significantly reduced its abundance. Diverse polymers such as Ethyl Vinyl Acetate (EVA), Styrene Allyl Alcohol (SAA) and Polytetrafluoroethylene (PTFE), which were not found in the other compartments, were found in the sediment. Cho et al.^62^ attributed the prevalence of PTFE in sediments to its high density.

Fragment-type particles were predominant across all compartments, with proportions of 89.2% at the surface, 70.8% in the subsurface, 59% in the water column, and 81.3% in the sediment (Figure 6). Additionally, a progressive decrease in fragment size with increasing depth was observed: 2.26 ± 1.38 mm at the surface, 2.10 ± 2.34 mm in the subsurface, 1.35 ± 0.94 mm in the water column, and 0.90 ± 0.49 mm in the sediment (Figure S4). This behavior is consistent with various studies that have reported a negative correlation between microplastic size and sampling depth in nearshore areas.^7,50^

**Figure 6:**
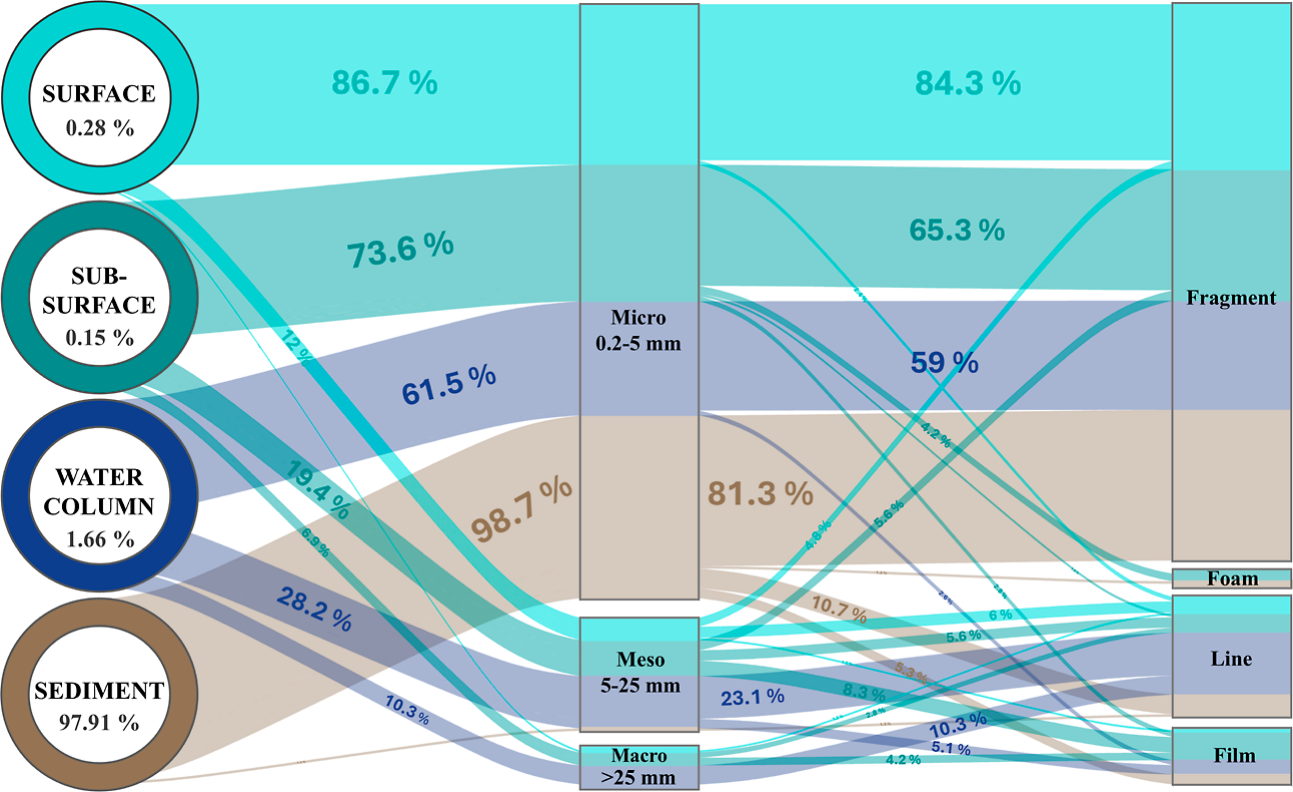
Conceptual flow diagram of the plastic composition in size (micro-, meso-, and macro-plastic) and shape category (fragment, foam, line, and film) for surface, subsurface, water column, and sediment. The percentages within the circles represent the data normalized by the depth range of each compartment, while the percentages in the flow diagram refer to the total number of plastics identified in each environment.

Despite the observed decrease in fragment size with increasing depth, an increment in the mean size of plastic particles was evident in the subsurface (5.75 ± 8.42 mm; minimum size 0.36 mm; maximum size 37.82 mm) and the water column (10.74 ± 16.97 mm; minimum size 0.36 mm; maximum size 76.11 mm). Indeed, the subsurface exhibited a predominance of microplastic particles (73.6%) alongside a higher proportion of mesoplastics (19.4%) and macroplastics (6.9%). Meanwhile, within the water column, the proportion of microplastics further decreased (61.5%), while the abundance of mesoplastics (28.2%) and macroplastics (10.3%) increased. This shift in plastic size was attributed to a higher prevalence of lines and films, with respective proportions of 9.7% and 15.3% in the subsurface and 33.3% and 7.7% in the water column, respectively. The presence of meso- and macroplastics in the water column is a singular finding, as existing records are scarce due to the limited number of studies and the common exclusion of plastics larger than 5 mm in the reported data. Lastly, the sediment was the compartment with the highest percentage of microplastic (98.7%), with only 1.3% corresponding to mesoplastic. Therefore, the mean size of the particles in the sediment decreased to 1.29 ± 1.89 mm (minimum size 0.27 mm; maximum size 15.64 mm), with the lowest standard deviation.

This variation in plastic composition has also been documented in nearshore areas.^63^ In our study, statistically significant differences were observed in both particle size (χ^2^ = 11.741, d*f* = 2, *p*-value = 0.0028) and shape (χ^2^ = 10.673, d*f* = 2, *p*-value = 0.0048) in relation to the distance from the coast. Post hoc pairwise comparisons using Bonferroni correction indicated significant differences between fragments and lines (adjusted *p*-value = 0.003), as well as between micro- and macroplastics (adjusted *p*-value = 0.015). In contrast, no significant differences were observed among polymer types (*p*-value >0.05). These results suggest that both particle shape and size may influence the spatial distribution of plastics, potentially driven by proximity to land-based sources.^64^

To further explore the mechanisms driving this distribution, the settling behavior of the plastic particles was assessed. The selective loss of particles from the surface layer to the rest of the water column is also affected by shape, size, and polymer density, thereby influencing both their rise and settling velocity.^65^ Indeed, the analysis of the settling velocities revealed clear trends associated with these factors (Table S7). Polymer density plays a critical role in controlling settling velocities; particles denser than the surrounding fluid exhibit higher velocities (mean 0.0109 m·s^–1^) compared to those less dense than seawater (mean 0.0145 m·s^–1^).

Particle shape also influenced settling dynamics, where films exhibited the lowest settling velocities, consistent with laboratory experiments attributing their slower descent to a higher drag coefficient.^15,66^ However, in the marine environment, this behavior may be altered by biofouling, which increases particle density and can accelerate sinking rates by up to 81%.^67,68^ This phenomenon may explain why film-type plastics are predominantly associated with coastal areas in subsurface waters, where they tend to settle rapidly due to biofouling.^30^ On the other hand, fragments represented the shape with the highest settling velocity, followed by lines. The primary difference was that fragments had a higher concentration at the surface than in deeper water layers, as they exhibited a rising velocity four times higher than lines.^69^ In contrast, lines persisted in the mixing zones for longer periods, which explained their relative predominance within the water column.

Particle size also influenced the settling behavior, with mean velocities progressively increasing with size. Although it remains challenging to establish the primary factor governing settling behavior, several studies have consistently identified polymer density and particle size as the most critical parameters controlling settling velocity.^70,71^ However, it is important to note that, while most existing settling velocity models are calibrated for microplastics (<5 mm), our findings suggest that future models should incorporate meso- and macro-plastic particles to more accurately represent the full dynamics of plastic transport across all size classes.

Furthermore, to assess the potential accumulation of plastics in different compartments, the total number of plastic particles was normalized to the depth range of each compartment. We found that the vast majority of plastics were located in the sediment, accounting for 97.91%. Meanwhile, the remaining particles were distributed in the water column (1.66%), surface (0.28%), and subsurface (0.15%). The consistency observed between the increase in the proportion of small microplastics (<1 mm) from the surface to deep layers (surface: 18.1%, subsurface: 41.2%, water column: 47.8%, and sediment: 70.9%) confirms the downward flux of these particles and their accumulation in the sediment. The size-dependent removal of smaller particles from the surface, as documented in several studies,^3,13,72^ reinforces the observed trends in our results. The enrichment of plastics within this size class in sediment further supports the conclusion that marine sediments serve as a major sink for plastic pollution, as highlighted by Martin et al.^73^ Nevertheless, it is important to consider that the exclusion of particles smaller than 200 μm may limit the interpretation of the vertical distribution and dynamics of small microplastics.

Overall, this study provides a comprehensive understanding of the vertical distribution of plastic particles in nearshore areas. The highest concentrations were observed in surface waters and sediments, with the latter serving as a long-term reservoir. Plastic concentration generally decreases with depth, although slight increases were detected in the deeper layers of the water column. However, understanding the vertical distribution pattern from the surface to the sediment remains complex due to the multitude of processes occurring within the water column. Our findings indicate that the vertical distribution of plastics is driven by the physical properties of the water column, which retain plastic particles in the mixed layer before reaching the pycnocline. Additionally, inherent particle characteristics—such as polymer density, shape, and size—play a significant role. Low-density polymers were found to dominate surface and subsurface waters (<1.17 m), whereas high-density polymers prevailed in deeper layers and sediments. Fragment-type particles were predominant across all compartments, while film- and line-type particles exhibited the highest proportions below the sea surface (>0.17 m), likely due to their susceptibility to biofouling and vertical transport. Microplastics represented the dominant size class in all compartments; however, their relative abundance decreased with increasing depth (>1.17 m).

Importantly, our results provide empirical depth-resolved data on plastic concentrations along with settling velocity estimates based on particle characteristics. These findings can improve plastic transport models by offering real-world constraints on the vertical particle distributions and behavior. Most existing models rely on assumptions derived from laboratory estimates or surface data extrapolations, which may not accurately reflect the in situ conditions. The vertical concentration gradients observed in our study—alongside polymer-specific settling behavior—can be integrated into modeling efforts to improve the representation of particle fluxes, vertical mixing, and retention in coastal systems. Additionally, the variability in plastic types and densities we observed highlights the need for particle-resolved modeling approaches that account for the shape, composition, and biofouling dynamics. Thus, our study not only complements existing modeling work but also fills in a critical data gap that can support the development of more accurate, spatially explicit simulations of plastic transport.

Nonetheless, we emphasize the need for further in situ studies combining high vertical resolution sampling, characterization of particle type and composition, and monitoring of physical forcing, including advection of different water layers. Hydrodynamic factors such as wind, tides, and waves can be key to understanding the vertical distribution of plastics in the nearshore areas. This knowledge is essential to understanding how plastic pollution behaves in the marine environment and to designing preventive and mitigation measures.

## Supplementary Material



## Notes

Photographs of instruments; additional details of sampling methods; categories of plastic particles; sediment sample processing; sediment concentration calculation; sampling details for Neuston Net, Bongo Net, Multinet, and Box Corer, including coordinates, filtered volume, depth, and plastic concentrations; Spearman’s rank correlation coefficients; data normalization for each compartment depth range; and description of the settling velocity model (PDF)
