# Supplementary material for: Vertical Distribution and Composition of Plastics in Coastal Areas of the Gulf of Cádiz: Insights into Transport Dynamics
Source: Environ Sci Technol. 2025 Aug 5;59(33):17760–72. doi: 10.1021/acs.est.5c03513 (PMC12392743; doi:10.1021/acs.est.5c03513)
Supplement: Supplementary file 1 [file es5c03513_si_001.pdf]

Supporting Information for

Vertical Distribution and Composition of Plastics in Coastal Areas of the Gulf of Cádiz: Insights into Transport Dynamics

Rocío Quintana[1*], Sandra Manzano-Medina[1], Lucía Pérez-López[1], Amets Oyón-Sanz[1], Daniel González-Fernández[1], Juan Ignacio González-Gordillo[1], Elisa Marti[1], Fidel Echevarría[1] and Carmen Morales-Caselles[1]

[1]Department of Biology, University Marine Research Institute INMAR, University of Cádiz and European University of the Seas SEA-EU, Puerto Real, Spain.

*Corresponding author.

Email: rocio.quintana@uca.es

Summary information:

Number of pages: 14

Paragraphs: 4

Figures: 4

Tables: 7

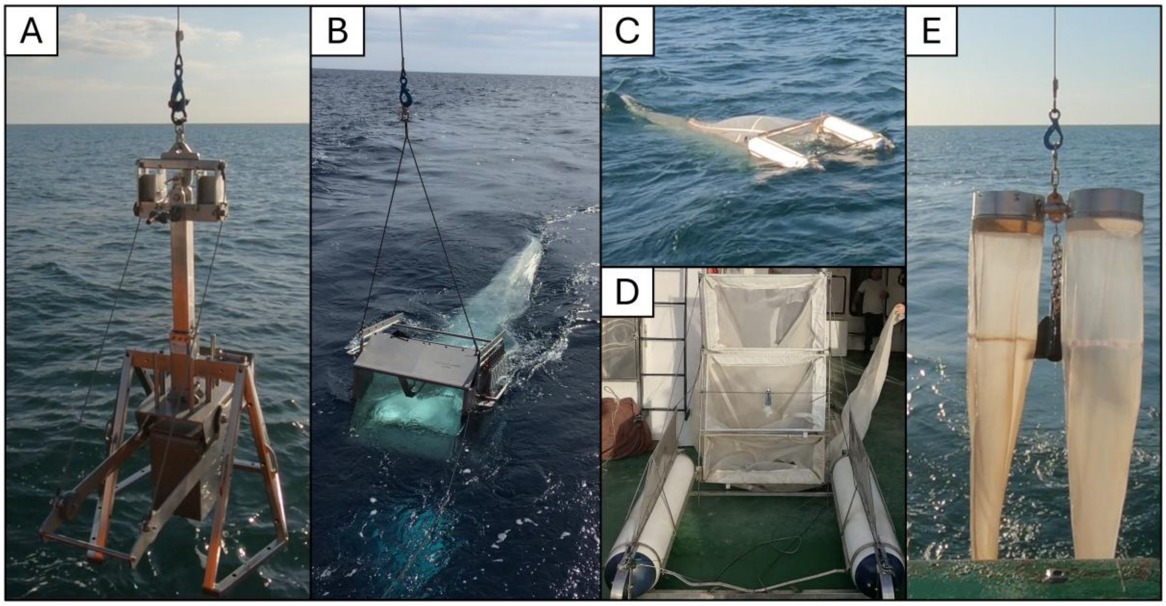

Figure S1. Instruments used in the campaign: (A) Box Corer, (B) Multinet, (C, D) Neuston Net and (E) Bongo Net.

Paragraph S1. Description of the sampling methods.

Surface and sub-surface water sampling was carried out with a modified neuston net furnished with three nets distributed consecutively on the vertical. The first net corresponding to the surface, has a mouth of 0.30 x 0.82 m, with an effective filtering area (opening area that is submerged) of 0.17 x 0.82 m (0.14 m^2). The other two nets, corresponding to the subsurface, have a mouth of 0.50 x 0.82 m (0.41 m^2). Water column sampling was performed with two different devices: a bongo net for shallow waters and a multiple plankton sampler for deeper waters. The bongo device is provided with two circular nets of 0.12 m^2 each. Both bongo net and neuston net are equipped with a General Oceanics flowmeter. The multiple plankton sampler (or also called Multinet - Hydrobios Multinet Mammoth, Kiel-Altenholz Germany) consisted of a total of nine individual nets attached to the stainless-steel frame with an aperture dimension of 1 m × 1 m. During each deployment, up to six water depths were sampled within the upper 100 m of the water column. The Multinet includes a CT-Set accessory located in the upper frame, and consists of a conductivity and temperature sensor, which computes salinity, density, and sound velocity according to UNESCO formulas [1]. The first net of the Multinet was used on the descent, from the surface till the bottom (5 meters above the sediment), to profile salinity and temperature (T/S) and obtain an integrated sample along the whole water column. Then, the five remaining nets were used on the ascent of the instrument and were opened at different depths according to the T/S profiles. All nets used in this campaign had a mesh size of 200 microns. Finally, sediment samples were collected using a box corer with a surface area of 0.105 m^2. The box corer was deployed three times at each sampling point to obtain three replicates. For each replicate, the top two centimeters were subsampled. All instruments, with the exception of the neuston net, were deployed from the stern over the A-frame of the R/V. The neuston net was deployed from the side of the

vessel, positioned outside the influence of the wake zone to avoid the effects of turbulence [2]. All nets were towed at a constant speed of 1.5 knots for approximately 10-15 minutes per deployment (for both the neuston net and the bongo net) or water depth sampling (for the Multinet). Meanwhile, for the box corer the R/V remained stationary for one hour.

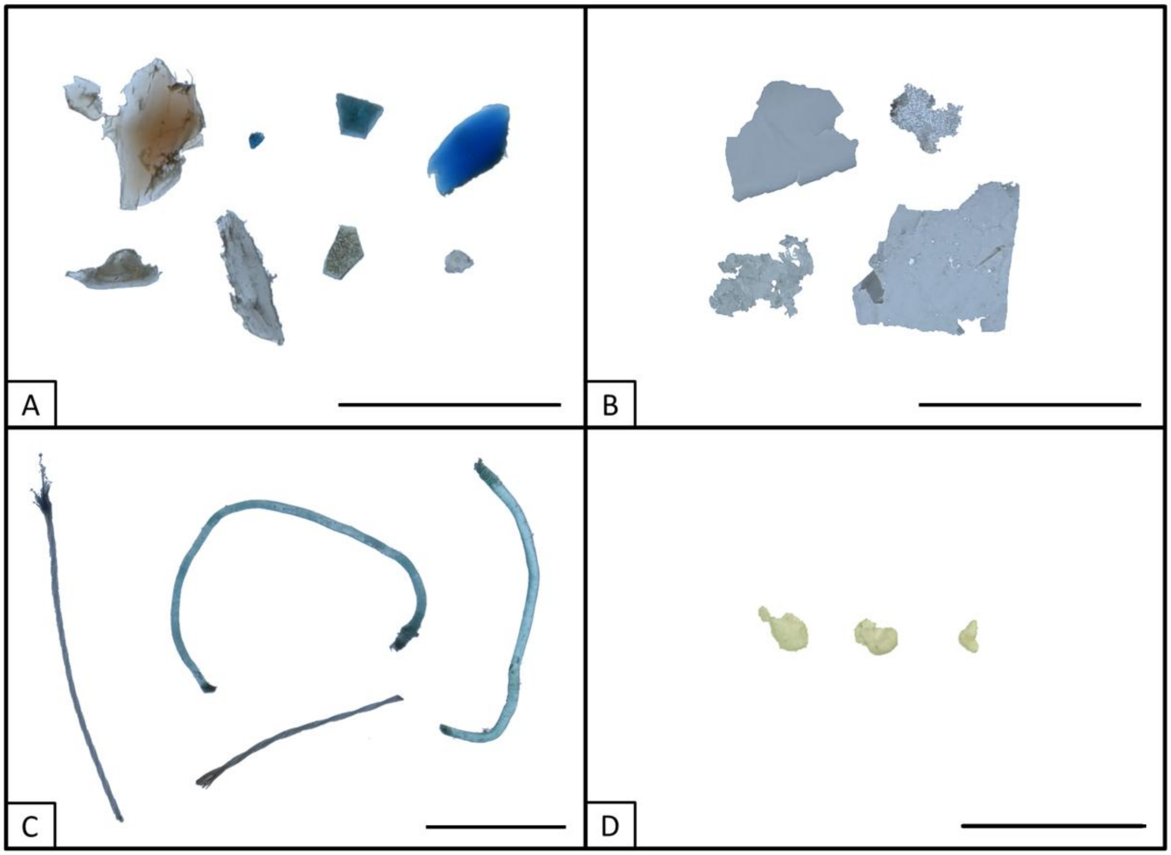

Figure S2. Categories of all the plastic particles found in the sample. (A) Fragment, (B) Film, (C) Line and (D) Foam. The scale below each picture corresponds to 5 mm.

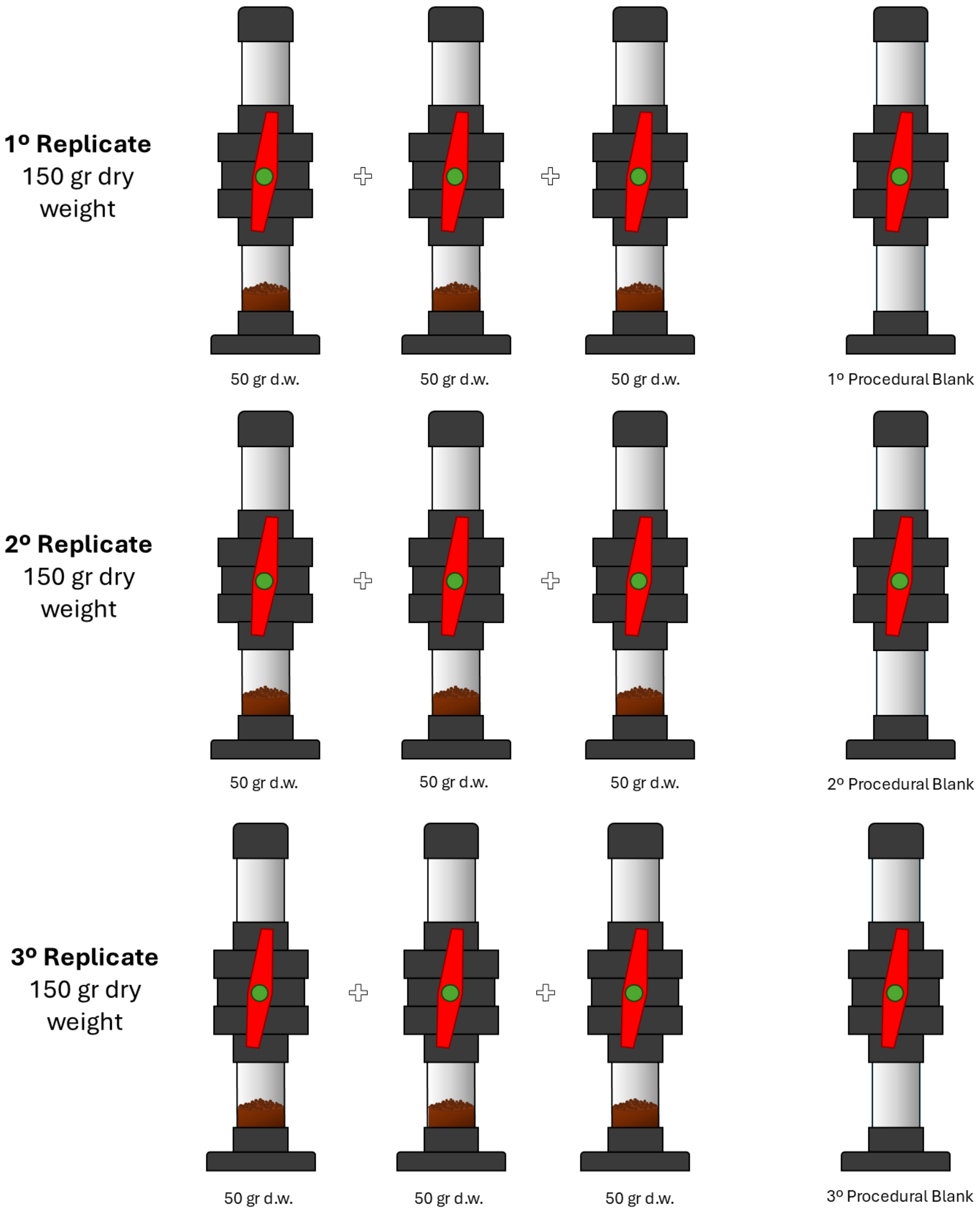

Figure S3. Schematic representation of the sediment sample extraction process for each replicate, including procedural blanks. Each replicate consists of 150 g dry weight (d.w.), divided into three 50 g portions processed sequentially. A blank control was performed after each replicate, following the same procedure without a sample.

Paragraph S2. Description of the sediment concentration calculation.

All sediment calculations are referenced to the dry weight (d.w.) of the sediment. To obtain the equivalence between wet and dry weight, approximately 8 grams of wet sediment were dried in 5 ml

tubes in a laboratory oven at 40ºC for five days. This process was repeated three times for each sediment replicate. Once the dry weight values were obtained, the equivalent to 50 grams of dry weight was calculated. These three values were averaged to establish a single value of 50 grams of dry weight with its associated standard deviation (minimum value: 62.2 g; maximum value: 154.7 g).

Plastic concentrations in sediment samples were calculated per sediment mass (items·kg^{-1}), surface area (items·m^{-2}), and volume (items·m^{-3}) using the following general equation:

$$C_w = \frac{n}{W_{dry}}$$

Where Cw is the concentration of microplastic, n represents the number of plastics per replicate and W is the dry weight (kg) of the sediment, which is a total of 150 gr of d.w.

Since the first two centimeters of the box corer were subsampled, the surface area will be referenced to those two centimeters, which have been previously homogenized. Sediment area was calculated using a core sampling method. First, wet sediment was added to the core and weighed to determine the volume:

$$V = \pi r^2 h$$

where V is the volume in m^3, r is the core radius, and h is the measured sediment height. To reference the results to 50 g d.w., we calculated the equivalent volume for 50 g by multiplying the total sediment volume by the ratio of dry to wet weight:

$$V_{50g} = V * \frac{W_{dry}}{W_{wet}}$$

Using this volume and considering the reference height of 2 centimeters, a new area was calculated and normalized to the equivalent of 50 g d.w. Thus:

$$A_{50g} = \frac{V_{50g}}{h_2}$$

With this new area (A_{50g}), concentrations were calculated per unit surface area:

$$C_A = \frac{n}{A_{50g}}$$

Finally, concentrations per volume were calculated:

$$C_p = \frac{n}{V_{50g}}$$

Table S1. Wet weight (g), area (m^2), and volume (cm^3) values used for each sediment sample replicate. All measurements refer to the equivalent of 150 g of dry sediment, including the corresponding area and volume.

	Station	Wet Weight (g)			Area (m^2)			Volume (cm^3)		
		Replicate 1	Replicate 2	Replicate 3	Replicate 1	Replicate 2	Replicate 3	Replicate 1	Replicate 2	Replicate 3
CÁDIZ	C1	234.9	250.2	209.8	0.006	0.007	0.005	124.8	146.2	103.3
	C2	322.4	349.1	408.9	0.011	0.013	0.014	216.4	258.6	274.3
	C3	414.7	421.7	457.0	0.014	0.016	0.016	283.8	327.2	318.4
	C4	407.3	284.5	223.4	0.014	0.009	0.006	289.5	187.5	123.9
	C5	385.1	388.3	394.2	0.012	0.013	0.013	234.8	266.8	259.3
	C6	419.4	380.9	401.7	0.016	0.014	0.014	310.5	281.4	279.7
	C7	248.3	342.6	396.7	0.008	0.010	0.014	150.4	193.3	286.0
GUADALQUIVIR	G1	398.4	274.5	387.6	0.014	0.007	0.013	275.2	131.1	267.8
	G2	368.1	376.1	379.8	0.012	0.014	0.014	239.7	272.6	270.7
	G3	395.6	387.1	392.1	0.014	0.013	0.013	274.9	252.3	266.1
	G4	192.5	204.0	204.1	0.005	0.005	0.005	96.1	109.6	108.6
	G5	187.2	215.7	207.4	0.004	0.005	0.005	87.6	109.3	107.2
	G6	367.4	373.9	376.0	0.013	0.015	0.013	251.7	306.3	261.5
	G7	207.0	198.7	206.5	0.005	0.007	0.006	96.4	131.7	123.1
	G8	286.8	279.7	231.8	0.009	0.009	0.006	184.1	184.1	121.6
	G9	251.1	263.2	257.2	0.008	0.008	0.008	152.2	161.0	155.4

Table S2. Information of each sample of the Neuston Net (surface and subsurface) and the Bongo Net (water column) with their respective coordinates, distance from coast (m), depth (m), volume (m^3) and abundance ($item\cdot m^{-3}$). ND (No Data) due to the loss of the net during the sampling.

			NEUSTON NET							BONGO NET					
	Station	Date	Coordinates		Dist (m)	Surface (0-0.17m)		Subsurface (0.17-1.17m)		Coordinates		Dist (m)	Water Column		
			Lat	Lon		Volume (m^3)	Abundance ($item\cdot m^{-3}$)	Volume (m^3)	Abundance ($item\cdot m^{-3}$)	Lat	Lon		Depth (m)	Volume (m^3)	Abundance ($item\cdot m^{-3}$)
CÁDIZ	C1	18/10/21	36.56	-6.35	4980	65.8	0.091	385.4	0.018	ND	ND	ND	ND	ND	ND
	C2	18/10/21	36.54	-6.41	9674	76.7	0	449.4	0.033	36.54	-6.43	11163	20	66.5	0.015
	C3	18/10/21	36.51	-6.51	18197	72.4	0.318	423.9	0.002	36.51	-6.50	16389	38	139.3	0
	C4	20/10/21	36.49	-6.33	4509	90.4	0.077	529.7	0.019	36.49	-6.34	5787	15	135.9	0
	C5	20/10/21	36.49	-6.37	7472	55.6	0.054	325.4	0	36.49	-6.36	6670	27	187.1	0.005
	C6	20/10/21	36.45	-6.46	16342	73.5	0.014	430.3	0.007	36.45	-6.45	16342	45	84.1	0.024
	C7	22/10/21	36.59	-6.49	10084	70.2	0.028	411.3	0.002	36.60	-6.48	8824	25	130.8	0
GUADALQUIVIR	G1	27/10/21	36.77	-6.47	4283	84.9	0.024	497.3	0.014	36.77	-6.46	4012	7	132.5	0.023
	G2	27/10/21	36.75	-6.51	5776	98.0	0	574.2	0.007	36.74	-6.52	6792	11	183.4	0.005
	G3	27/10/21	36.70	-6.57	12073	83.0	0.012	486.0	0	36.69	-6.57	11680	20	123.1	0
	G4	26/10/21	36.83	-6.47	5420	85.5	0	500.8	0.002	36.82	-6.47	5456	7	161.7	0.006
	G5	26/10/21	36.80	-6.53	11190	83.1	0.060	486.6	0.004	36.81	-6.52	10431	10	260.1	0.008
	G6	26/10/21	36.79	-6.57	14584	85.7	0	501.9	0.002	36.79	-6.57	15453	16	170.4	0.006
	G7	26/10/21	36.86	-6.47	3913	97.2	0.021	569.1	0.002	36.86	-6.48	4453	4	126.2	0
	G8	26/10/21	36.85	-6.54	9790	91.6	0.109	536.6	0.004	36.85	-6.53	9103	10	149.6	0
	G9	26/10/21	36.84	-6.61	16010	95.8	0.073	561.3	0	36.84	-6.61	16284	15	148.0	0.007

Table S3. Information of each throw of the Multinet and Neuston net with their respective coordinates, distance to the coast (m), depth (m), volume (m^3), and plastic concentration ($item \cdot m^{-3}$). ND (No Data) due to the loss of the net during the sampling.

	Station	Date	Lat	Lon	Distance (m)	Instrument	Net	Depth (m)	Volume (m^3)	Abundance ($item \cdot m^{-3}$)
CÁDIZ	MC1	18/10/21	36.51	-6.51	18197	Neuston Net	1	0 - 0.17	72	0.318
							2	0.17 - 0.67	212	0.005
							3	0.67 - 1.17	212	0
			36.52	-6.51	18453	Multinet	1	27.9 - 40.8	298	0.023
							2	16.3 - 28.3	199	0.005
							3	15.7 - 16.9	194	0.010
							4	0 - 16.6	107	0.019
	MC2	20/10/21	36.43	-6.62	30034	Multinet	1	0.1 - 85.3	512	0.004
							2	67.6 - 86.3	205	0
							3	48.5 - 67.2	153	0
							4	35.5 - 48.4	153	0
							5	26.1 - 35.2	169	0.006
							6	0.9 - 26.5	171	0.029
	MC3	22/10/21	36.50	-6.70	30914	Neuston Net	1	0 - 0.17	86	0.023
							2	0.17 - 0.67	251	0.008
							3	0.67 - 1.17	ND	ND
			36.50	-6.70	31091	Multinet	1	1.5 - 94	303	0.003
							2	68.9 - 94.2	272	0.004
							3	48.1 - 68.9	224	0
							4	34.1 - 48.3	188	0
							5	23.1 - 34.2	151	0.007
							6	8.3 - 23.3	183	0.005
GUADALQUIVIR	MG4	25/10/21	36.64	-6.79	32245	Neuston Net	1	0 - 0.17	94	0.032
							2	0.17 - 0.67	274	0.007
							3	0.67 - 1.17	274	0.015
			36.66	-6.81	34044	Multinet	1	3.2 - 97.1	531	0
							2	70.2 - 96.9	234	0
							3	51.8 - 69.8	208	0
							4	35.4 - 51.7	149	0.007
							5	24.8 - 36.5	76	0
							6	9.4 - 24.6	125	0.008
	MG5	25/10/21	36.72	-6.88	39278	Neuston Net	1	0 - 0.17	115	0.061
							2	0.17 - 0.67	337	0.009
							3	0.67 - 1.17	337	0.003
			36.71	-6.86	37972	Multinet	1	35.9 - 98	262	0.004
							2	69.1 - 101.1	297	0
							3	49 - 69.3	211	0
							4	31.5 - 48.8	202	0
							5	19.8 - 31.5	215	0
							6	9.8 - 19.8	131	0

Table S4. Information of each throw of the Box Corer with their respective coordinates, distance from coast (m), depth (m), and abundance (items·m^{-2}, items·kg^{-1} and items·m^{-3}). S.D. is the standard deviation of each sample due to the triplicates.

	BOX CORER												
	Station	Date	Coordinates		Distance (m)	Depth (m)	Sediment					
			Lat	Lon			Abundance (items·m^{-2})	S.D.	Abundance (items·kg^{-1})	S.D.	Abundance (items·m^{-3})	S.D.
CÁDIZ	C1	18/10/21	36.56	-6.35	4280	20	346.1	223.2	13.33	6.67	17303.7	11158.0
CÁDIZ	C2	18/10/21	36.54	-6.42	9030	27	56.6	49.6	4.44	3.85	2829.4	2479.2
CÁDIZ	C3	18/10/21	36.51	-6.49	15455	55	85.7	34.9	8.89	3.85	4287.2	1743.1
CÁDIZ	C4	20/10/21	36.50	-6.33	8091	20	0	0	0	0	0	0
CÁDIZ	C5	20/10/21	36.49	-6.38	14842	35	25.0	43.3	2.22	3.85	1249.3	2163.8
CÁDIZ	C6	20/10/21	36.45	-6.44	21821	53	47.4	82.1	4.44	7.70	2368.9	4103.0
CÁDIZ	C7	22/10/21	36.60	-6.47	7674	29	104.4	104.9	8.89	10.18	5220.8	5244.1
GUADALQUIVIR	G1	27/10/21	36.77	-6.46	7019	10	49.1	42.6	4.44	3.85	2456.2	2127.7
GUADALQUIVIR	G2	27/10/21	36.74	-6.51	12602	14	52.3	45.6	4.44	3.85	2613.8	2277.6
GUADALQUIVIR	G3	27/10/21	36.70	-6.58	20177	24	99.2	110.5	8.89	10.18	4958.5	5523.6
GUADALQUIVIR	G4	26/10/21	36.83	-6.47	5115	9	208.2	360.6	6.67	11.55	10408.3	18027.7
GUADALQUIVIR	G5	26/10/21	36.81	-6.51	9303	13	1484.1	1338.5	46.67	37.12	74202.8	66922.6
GUADALQUIVIR	G6	26/10/21	36.79	-6.58	15925	18	77.5	76.5	6.67	6.67	3874.1	3825.7
GUADALQUIVIR	G7	26/10/21	36.86	-6.47	3876	10	256.6	314.5	11.11	13.88	12831.6	15723.5
GUADALQUIVIR	G8	26/10/21	36.85	-6.52	8027	13	127.2	32.2	6.67	0	6362.1	1612.3
GUADALQUIVIR	G9	26/10/21	36.83	-6.61	16792	18	129.0	131.4	6.67	6.67	6449.6	6572.3

Table S5. Spearman's rank correlation coefficients (ρ) and p-values between plastic concentration and distance for the overall dataset and by study area (Cádiz and Guadalquivir) across different compartments.

Compartment	Overall		Cádiz		Guadalquivir	
	ρ	p-value	ρ	p-value	ρ	p-value
**Surface**	0.095	0.727	-0.107	0.840	0.153	0.695
**Subsurface**	-0.400	0.124	-0.396	0.379	-0.539	0.135
**Water Column**	0.127	0.651	0.273	0.600	-0.009	0.983
**Sediment**	-0.388	0.138	-0.464	0.302	-0.300	0.437

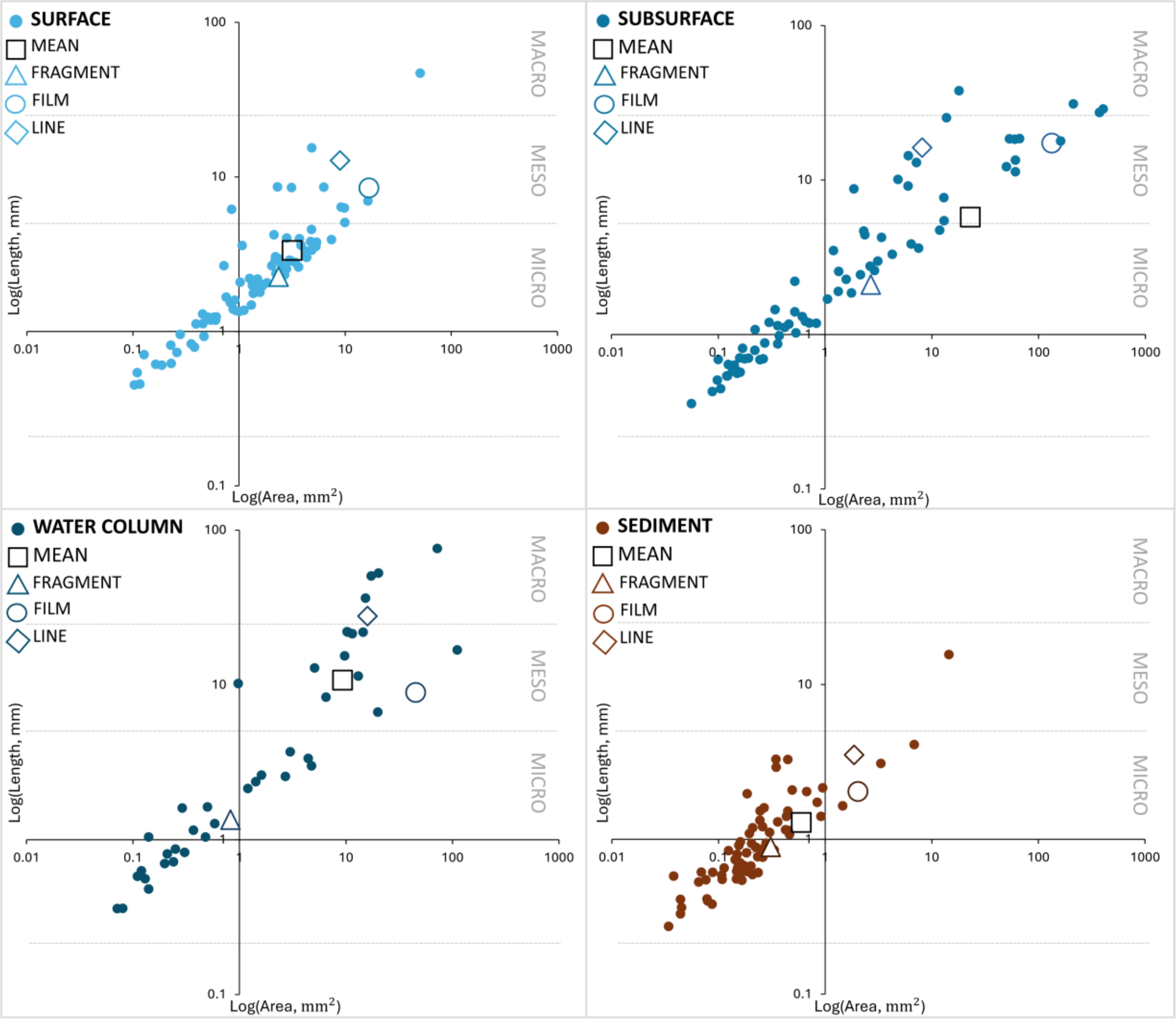

Figure S4. Area (mm²) and length (mm) of all the plastic particles that were found in this study in the surface, subsurface, water column and sediment. The area and length are in log scale for the comparison with other size ranges. The mean values for the total particle count and each particle type (fragment, film, and line) are included. Horizontal grey dotted lines indicate the size fraction: microplastic (0.2-5 mm), mesoplastic (5-25 mm) and macroplastic (>25 mm).

Paragraph S3. Data normalization of each compartment depth range.

To compare the distribution of plastic particles across compartments, the concentrations of plastics obtained were normalized by the corresponding depth range of each compartment using the following equation:

$$N_{normalized} = C * h$$

where $N_{normalized}$ represents the normalized particle count per unit area (items·m^{-2}), C is the average concentration for each compartment (items·m^{-3}), and h is the depth range of the compartment (m) referring to the vertical extent of each sampled layer. The proportion of particles in each compartment was then expressed as a percentage of the total using the formula:

$$Proportion\ (\%) = \frac{N_{normalized}}{N_{total}} * 100$$

This normalization approach allowed for the direct comparison of particle distributions between compartments with varying depth ranges, providing a standardized representation of the vertical distribution of plastic particles.

Paragraph S4. Description of the settling velocity model.

The settling velocity model used in this study is from Yu et al. [3], as it represents a more recent approach and has been identified as the most appropriate settling velocity model in the context of microplastic transport, according to a comparative study by Coyle et al. [4]. This model describes the vertical transport of plastics under the influence of gravity, buoyancy, and drag forces in a quiescent water column. Processes such as wind mixing, biofouling, incorporation into aggregates, and fecal pellets, which can also influence vertical transport, were not included in this analysis.

First, all plastic particles were analyzed using the software ImageJ Fiji, which provided a two-dimensional (2D) representation of each particle. From these images, the maximum length of the particle (also referred to as the Feret diameter) was obtained.

The Corey Shape Factor (CSF) was calculated using the following equation:

$$CSF = \frac{c}{\sqrt{ab}}$$

Where a represents the longest axis length of the particle, b the intermediate axis length, and c the shortest axis length (thickness). Since the analysis was performed using 2D images, only two axes (a and b) could be directly measured. The assignment of axes was dependent on the particle category (e.g., fragment, line, or film). Foam particles were excluded from the settling velocity analysis due to their limited presence in this study (n=3).

- For line particles, the a-axis corresponded to the maximum length, while b and c were assumed to be equal and represented the minimum length of the particle.
- For film particles, the a- and b-axes corresponded to the maximum and intermediate lengths obtained from the 2D image, respectively, while the c-axis (thickness) was assigned a constant value of 0.01 mm.

- For fragment particles, a similar proportion as that of line particles was applied; however, in this case, the b- and c-axes represented the intermediate dimension.

The volume-equivalent sphere diameter d_p is calculated following the next equation:

$$d_p = \sqrt[3]{\frac{6}{\pi} V_p}$$

Where Vp is the volume of the particle, that is obtained from the weight (g) and density ($g \cdot cm^{-3}$) of the particle. Plastic weights were measured using an OHAUS Explorer™ Semi-Micro Balance (EX225D/AD), with a readability of 0.01 mg, a repeatability of ± 0.015 mg, and a linearity of ± 0.02 mg. The balance was regularly calibrated using the built-in AutoCal™ system to ensure measurement accuracy. Particles densities were obtained from Table S5, and the mean density value for each polymer was used in the calculations. The dimensionless particle diameter (d_*) is defined as:

$$d_* = \left[\frac{(\rho_p - \rho_f) g}{\rho_f {\nu_f}^2}\right]^{\frac{1}{3}} d_p$$

Where g is acceleration of gravity, ν_f is the kinematic viscosity of the fluid ($m^2 \cdot s^{-1}$), ρ_p is the density of the particle and ρ_f is the density of the fluid. ν_f is obtained by the following equation:

$$\nu_f = \frac{\mu_f}{\rho_f}$$

Where and μ_f is the dynamic viscosity of the fluid. The dynamic viscosity was obtained from the temperature and density of the CT-set of the Multinet. Then it was calculated following the equations of ITTC [5]. $C_{d,s}$ is the drag coefficient for spherical particles proposed by Cheng [6]:

$$C_{d,s} = \frac{432}{{d_*}^3}(1 + 0.022{d_*}^3)^{0.54} + 0.47[1 - \exp(-0.15{d_*}^{0.45})]$$

That allowed us to calculate the drag coefficient C_d (dimensionless) proposed by Yu et al. [3]:

$$C_d = \frac{C_{d,s}}{({d_*}^{\beta_1} \phi^{{d_*}^{\beta_2}} CSF^{{d_*}^{\beta_3}})^{\beta_4}}$$

Where β_1, β_2, β_3 and β_4 are constants ($\beta_1 = -0.25$, $\beta_2 = 0.03$, $\beta_3 = 0.33$ and $\beta_4 = 0.25$) and Φ is the particle sphericity. The settling velocity ($m \cdot s^{-1}$) of irregularly shaped particles is defined as:

$$w_s = \left(\nu_f g \frac{\rho_p - \rho_f}{\rho_f}\right)^{\frac{1}{3}} \sqrt{\frac{4 d_*}{3 C_d}}$$

Only plastic particles smaller than 5 mm were considered in the settling velocity calculations, as the equations applied are specifically developed for this size range.

Table S6. Minimum, maximum and mean density of the polymers.

Polymer	Minimum Density (g·cm^{-3})	Maximum Density (g·cm^{-3})	Mean Density (g·cm^{-3})	Reference
PP	0.85	0.92	0.88	7
PE	0.89	0.98	0.93	
EVA	0.92	0.95	0.93	
PDMS	0.97	0.97	0.97	
PS	1.04	1.04	1.04	
PA	1.12	1.15	1.13	
PC	1.20	1.22	1.21	
PU	1.20	1.26	1.23	
PVC	1.38	1.41	1.39	
PTFE	2.10	2.30	2.20	
ABS-PVC	1.04	1.41	1.22	
PVA	1.29	1.29	1.29	8
PET	1.34	1.39	1.36	
PEA	1.12	1.12	1.12	9
MUF	1.29	1.29	1.29	10
SAA	1.08	1.08	1.08	11
PVS	0.98	0.98	0.98	
SBC	1.04	1.04	1.04	

Table S7. Median and mean settling velocities (m·s^{-1}) according to the particle shape (fragment, film and line), size, main polymer type, and buoyancy (ρ_p: particle density; ρ_f: seawater density ~1.025 g/cm³). S.D. is the standard deviation.

Composition		Median	Mean	S.D.
**Shape**	Fragment	0.0107	0.0119	0.0076
	Line	0.0118	0.0104	0.0041
	Film	0.0033	0.0036	0.0018
**Size**	0.2 – 1 mm	0.0057	0.0069	0.0054
	1 – 2 mm	0.0125	0.0123	0.0054
	2 – 3 mm	0.0178	0.0170	0.0079
	3 – 4 mm	0.0206	0.0185	0.0077
	4 – 5 mm	0.0147	0.0153	0.0053
**Polymer**	PE	0.0114	0.0118	0.0060
	PP	0.0087	0.0107	0.0066
	PET	0.0148	0.0174	0.0090
	PVC	0.0253	0.0228	0.0149
**Buoyancy**	Positive ($\rho_p < \rho_f$)	0.0098	0.0109	0.0063
	Negative ($\rho_p > \rho_f$)	0.0120	0.0145	0.0109

References

(1) IOC; SCOR; IAPSO. The International Thermodynamic Equation of Seawater – 2010: Calculation and Use of Thermodynamic Properties.; 2010.
(2) Kovač Viršek, M.; Palatinus, A.; Koren, Š.; Peterlin, M.; Horvat, P.; Kržan, A. Protocol for Microplastics Sampling on the Sea Surface and Sample Analysis. J. Vis. Exp. **2016**, No. 118, 55161. https://doi.org/10.3791/55161-v.
(3) Yu, Z.; Yang, G.; Zhang, W. A New Model for the Terminal Settling Velocity of Microplastics. Mar. Pollut. Bull. **2022**, 176. https://doi.org/10.1016/j.marpolbul.2022.113449.
(4) Coyle, R.; Service, M.; Witte, U.; Hardiman, G.; McKinley, J. Modeling Microplastic Transport in the Marine Environment: Testing Empirical Models of Particle Terminal Sinking Velocity for Irregularly Shaped Particles. ACS ES T Water **2023**, 3 (4), 984–995. https://doi.org/10.1021/acsestwater.2c00466.
(5) ITTC. Fresh Water and Seawater Properties. **2011**.
(6) Cheng, N.-S. Comparison of Formulas for Drag Coefficient and Settling Velocity of Spherical Particles. Powder Technol. **2009**, 189 (3), 395–398. https://doi.org/10.1016/j.powtec.2008.07.006.
(7) Erni-Cassola, G.; Zadjelovic, V.; Gibson, M. I.; Christie-Oleza, J. A. Distribution of Plastic Polymer Types in the Marine Environment; A Meta-Analysis. J. Hazard. Mater. **2019**, 369, 691–698. https://doi.org/10.1016/j.jhazmat.2019.02.067.
(8) Freshwater Microplastics: Emerging Environmental Contaminants?; Wagner, M., Lambert, S., Eds.; The Handbook of Environmental Chemistry; Springer International Publishing: Cham, 2018; Vol. 58. https://doi.org/10.1007/978-3-319-61615-5.
(9) Andreozzi, L.; Castelvetro, V.; Faetti, M.; Giordano, M.; Zulli, F. Rheological and Thermal Properties of Narrow Distribution Poly(Ethyl Acrylate)s. Macromolecules **2006**, 39 (5), 1880–1889. https://doi.org/10.1021/ma052190+.
(10) Antunes, A.; Duarte, M.; Paiva, N.; Ferra, J.; Martins, J.; Carvalho, L.; Barros-Timmons, A.; Magalhães, F. D. Partial Replacement of Melamine by Benzoguanamine in MUF Resins towards Improved Flexibility of Agglomerated Cork Panels. Int. J. Adhes. Adhes. **2018**, 87, 142–150. https://doi.org/10.1016/j.ijadhadh.2018.10.004.
(11) Yuan, Z.; Nag, R.; Cummins, E. Ranking of Potential Hazards from Microplastics Polymers in the Marine Environment. J. Hazard. Mater. **2022**, 429, 128399. https://doi.org/10.1016/j.jhazmat.2022.128399.
